# Supplementary material for: Flavivirus maturation leads to the formation of an occupied lipid pocket in the surface glycoproteins
Source: Nat Commun. 2021 Feb 23;12:1238. doi: 10.1038/s41467-021-21505-9 (PMC7902656; doi:10.1038/s41467-021-21505-9)
Supplement: Supplementary file 1 — Supplementary Information [file 41467_2021_21505_MOESM1_ESM.pdf]

Supplementary Information

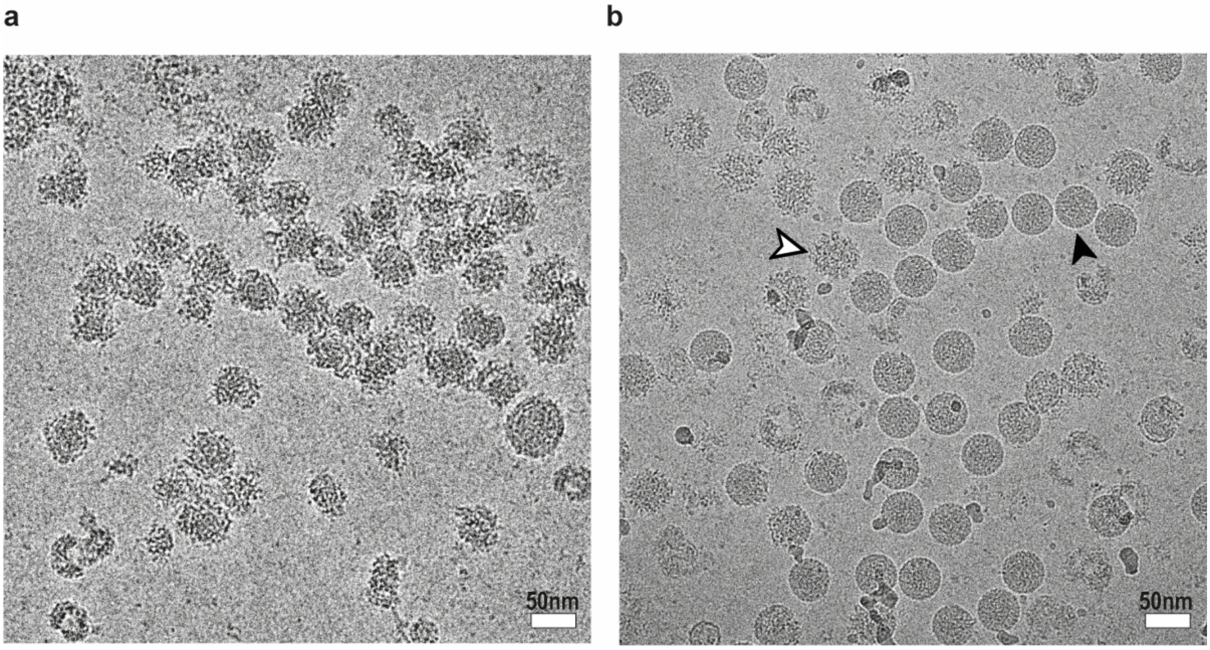

Supplementary Figure 1. UV inactivation procedure. a, Micrograph of inactivated SPOV particles after 5 minutes of UV exposure. The shown micrograph is a representative of a dataset containing a total of 5712 micrographs. The particles are heavily distorted and aggregated. White bar ~50nm. **b**, Micrograph after optimized UV inactivation. The shown micrograph is a representative of a dataset containing a total of 15372 micrographs. Virus was exposed to UV light for varying periods of time (see Methods), followed by plaque assay to assess viricidal effect. The minimum exposure time of 1 min was identified, after which no plaques could be detected. For cryo-EM samples, we used an exposure time of 2 mins. The particles are of high quality and well-separated. Both mature particles (black arrow) as well as immature particles (white arrow) are present in the sample. White bar ~50nm.

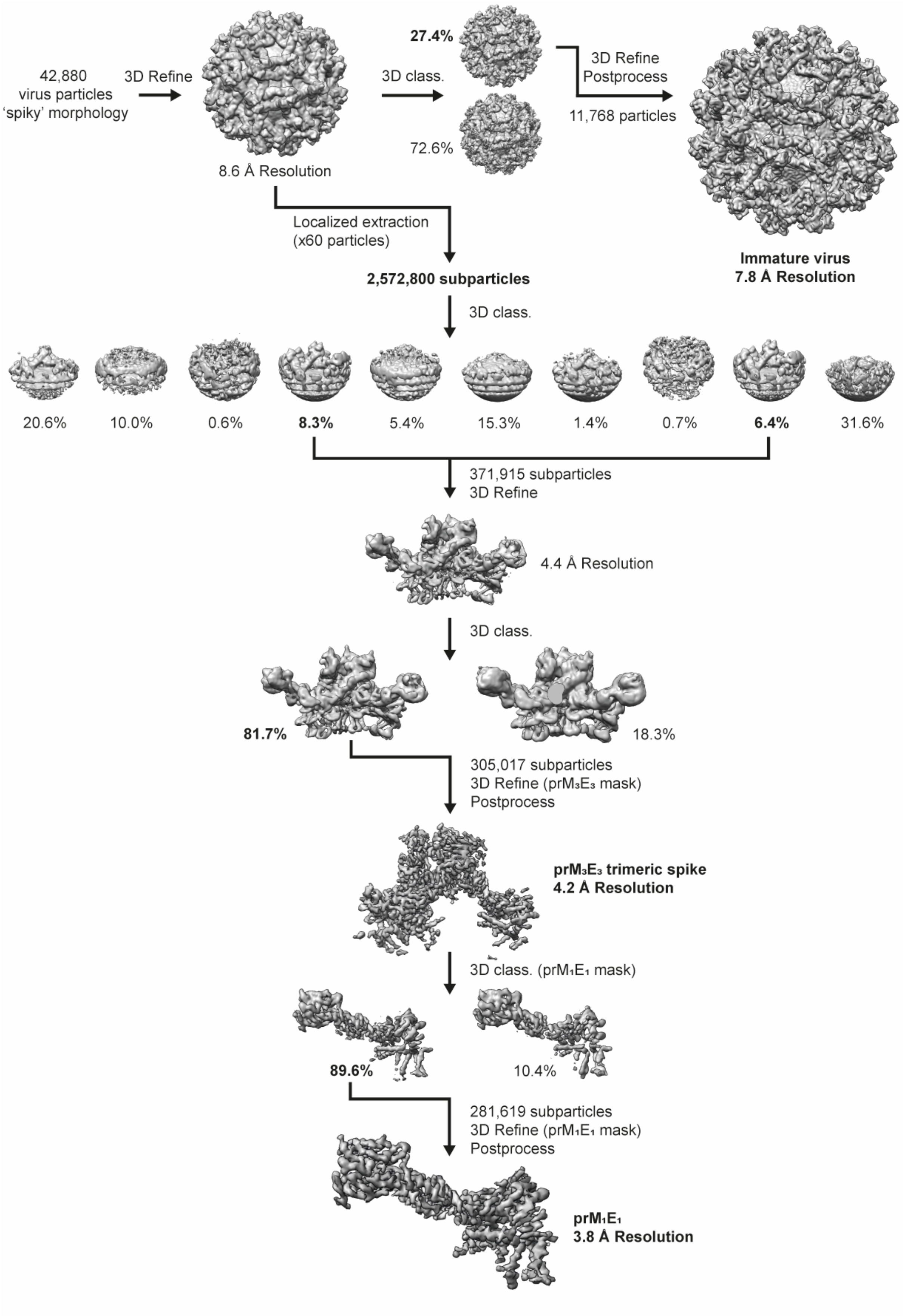
42,880
virus particles
'spiky' morphology
3D Refine
8.6 Å Resolution
3D class.
27.4%
72.6%
3D Refine
Postprocess
11,768 particles
Immature virus
7.8 Å Resolution
Localized extraction
(x60 particles)
2,572,800 subparticles
3D class.
20.6%
10.0%
0.6%
8.3%
5.4%
15.3%
1.4%
0.7%
6.4%
31.6%
371,915 subparticles
3D Refine
4.4 Å Resolution
3D class.
81.7%
18.3%
305,017 subparticles
3D Refine (prM3E3 mask)
Postprocess
prM3E3 trimeric spike
4.2 Å Resolution
3D class. (prM1E1 mask)
89.6%
10.4%
281,619 subparticles
3D Refine (prM1E1 mask)
Postprocess
prM1E1
3.8 Å Resolution

Supplementary Figure 2. Cryo-EM processing flowchart of immature SPOV. Flowchart of processing steps of immature SPOV, going from immature virions, to localized reconstruction of trimeric spikes, to focussed refinement of prM_1E_1 monomers.

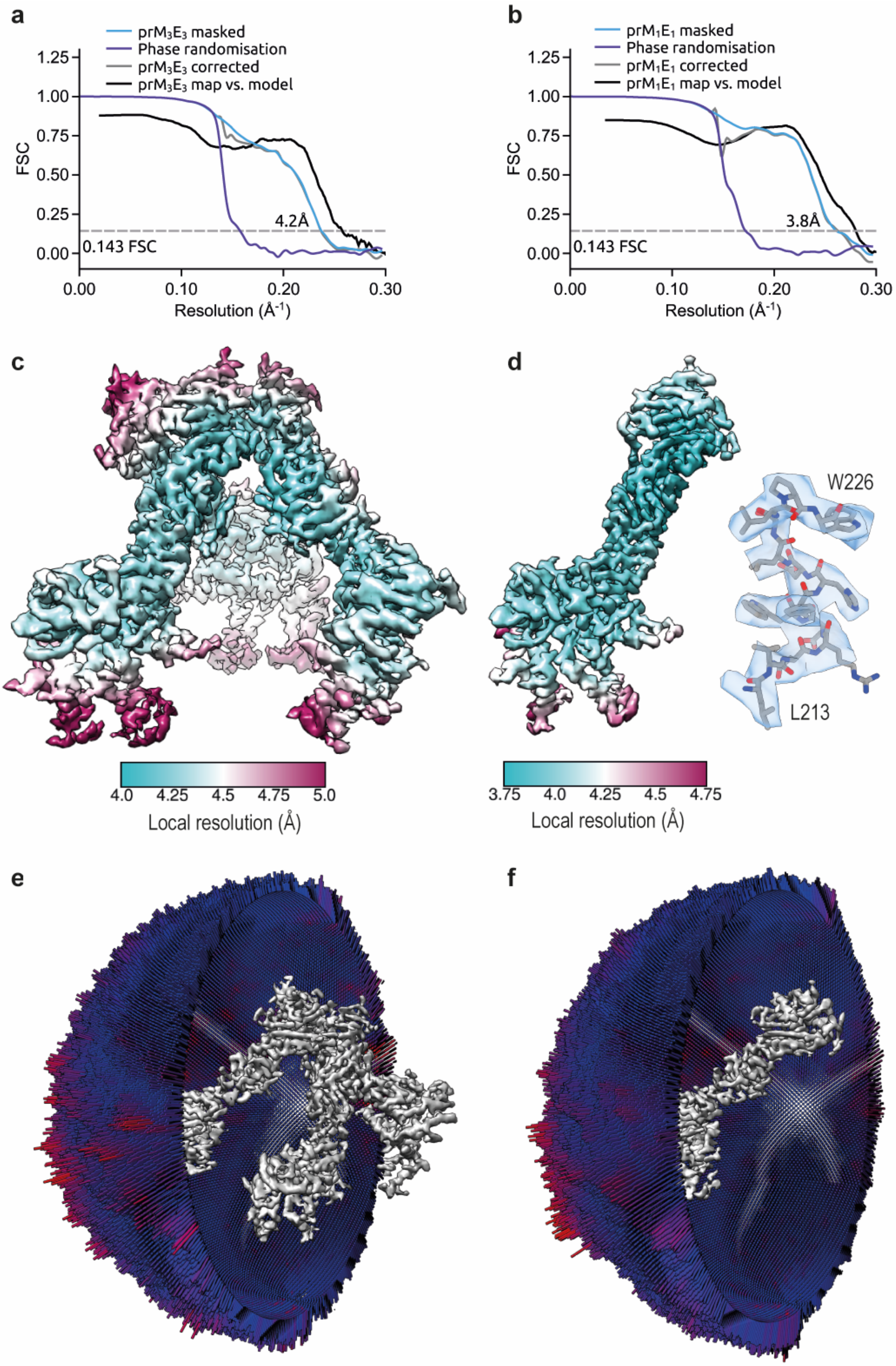
a
prM3E3 masked
Phase randomisation
prM3E3 corrected
prM3E3 map vs. model
FSC
4.2Å
0.143 FSC
Resolution (Å-1)
b
prM1E1 masked
Phase randomisation
prM1E1 corrected
prM1E1 map vs. model
FSC
3.8Å
0.143 FSC
Resolution (Å-1)
c
4.0 4.25 4.5 4.75 5.0
Local resolution (Å)
d
W226
L213
3.75 4.0 4.25 4.5 4.75
Local resolution (Å)
e
f

Supplementary Figure 3. Cryo-EM localized reconstruction and model refinement of immature SPOV. a, and **b**, Fourier shell correlation (FSC) plots of reconstructions using gold-standard refinement in RELION for prM_3E_3 trimers and prM_1E_1 monomer, respectively. Approximate map resolutions according to the 0.143 FSC cutoff are indicated for all reconstructions. Curves are shown for masked maps, phase randomization, corrected maps, and map vs refined atomic model. **c**, and **d**, 3D reconstructions of trimer and monomer, coloured by local resolution (according to RELION). In panel d, a representative fit of model to density is shown. **e**, and **f**, Angular distribution of particles for the reconstructions of trimer and monomer, respectively.

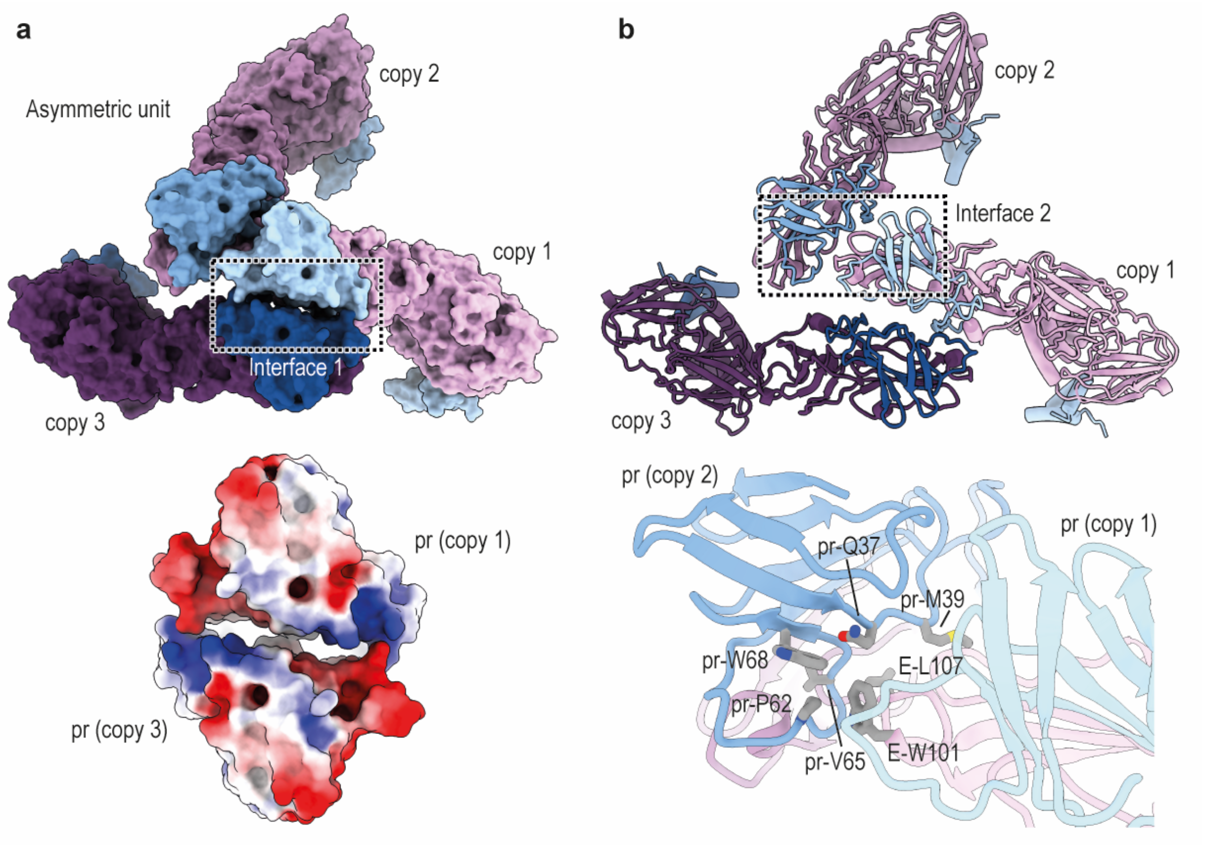

Supplementary Figure 4. Interaction surfaces within prM_3E_3 spikes. a, Topview of a prM_3E_3 trimeric spike, shown in surface representation (prM copies are coloured in shades of blue, E in shades of purple). The trimer represents the content of an icosahedral asymmetric unit of the virus. The three copies of prM_1E_1 are indicated. Intra-spike interaction is primarily mediated through the pr-domains. The bottom of the panel shows a close-up of the electrostatic surface (coloured from blue for positively charged to red for negatively charged) of the indicated interface 1. Complementary positively and negatively charged surfaces are responsible for the interaction between pr of copy 1 and pr of copy 3. **b**, Topview of the trimer shown in the same orientation as in panel a, but in ribbon representation. The second major interaction interface is indicated and a close up of the interface is shown at the bottom of the panel. Interface 2 is dominated by hydrophobic contacts. Involved residues are indicated.

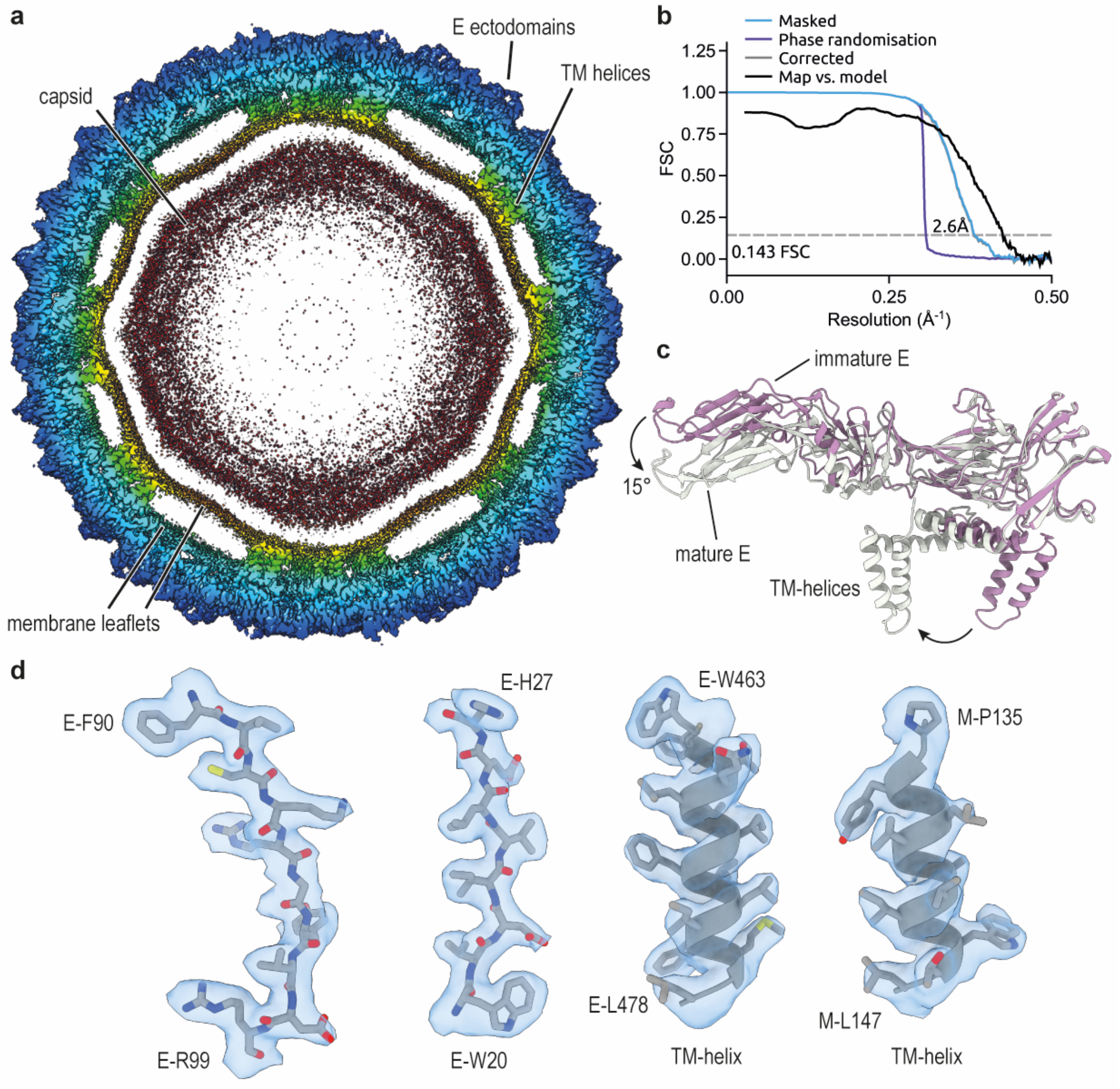

Supplementary Figure 5. Icosahedral cryo-EM reconstruction and model refinement of mature SPOV. a, Slice through the cryo-EM density of the virion, coloured by radius from red to blue. **b**, Fourier shell correlation (FSC) plots using gold-standard refinement in RELION. Approximate map resolution according to the 0.143 FSC cutoff is indicated. Curves are shown for masked maps, phase randomization, corrected maps, and map vs refined atomic model. **c**, Hinge motion of the E protein. Aligned ectodomains of E reveal a ~15° hinge movement as indicated (mature E in grey, immature E in purple). Also of note is the repositioning of the membrane associated helices, going from immature to mature E. **d**, Close-ups of cryo-EM density with atomic model, showcasing the quality of the map. TM-helices are also shown for E and M.

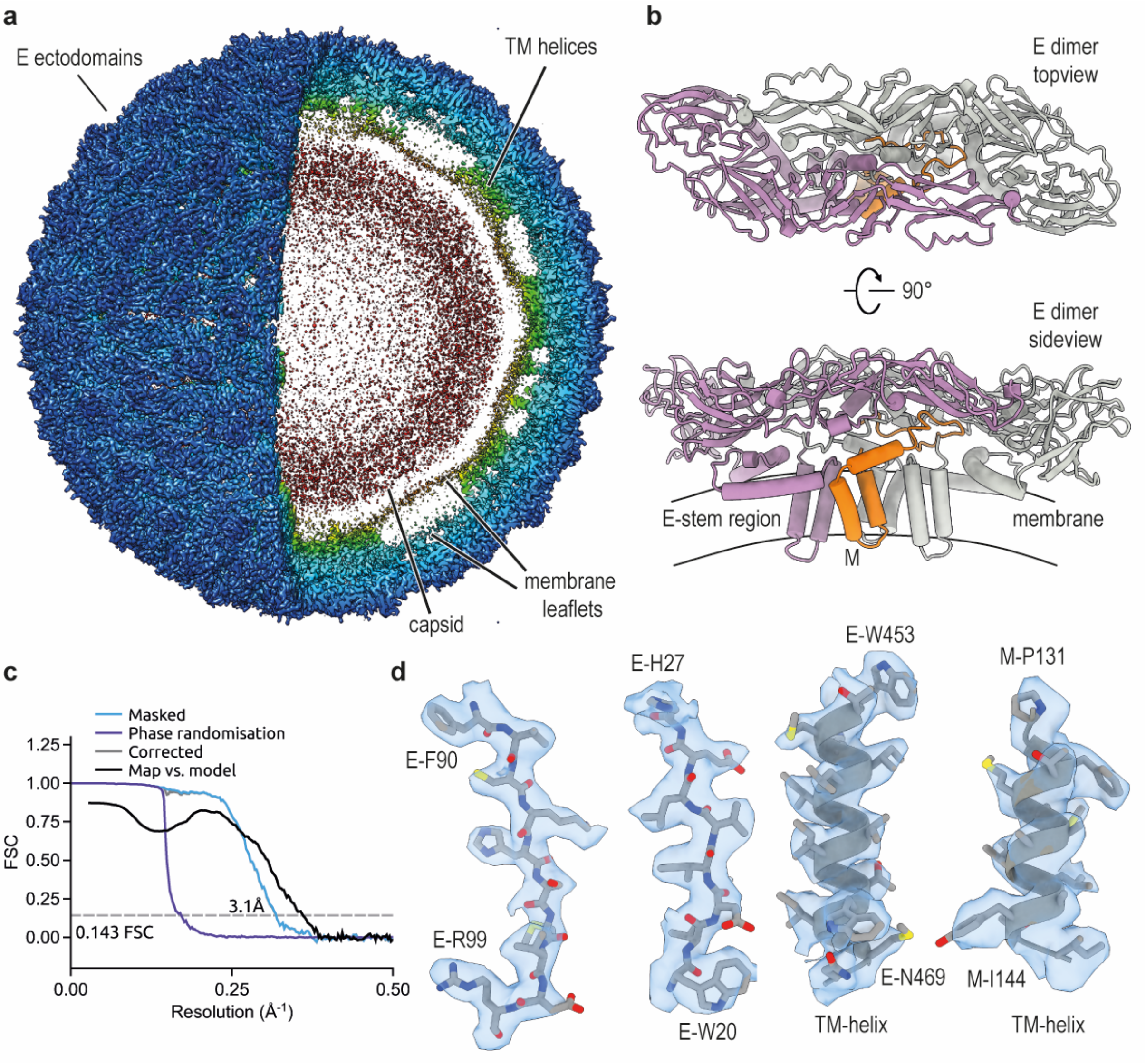

Supplementary Figure 6. Icosahedral cryo-EM reconstruction and model refinement of mature DENV2. **a**, Surface and sliced view of the cryo-EM density of the virion, coloured by radius from red to blue. **b**, Top-view and side-view of an antiparallel dimer of E, which compose the surface of the mature virus, in ribbon representation. The M protein is indicated in the side-view, as well as a schematic of the viral membrane. **c**, Fourier shell correlation (FSC) plots using gold-standard refinement in RELION. Approximate map resolution according to the 0.143 FSC cutoff is indicated. Curves are shown for masked maps, phase randomization, corrected maps, and map vs refined atomic model. **d**, Close-ups of cryo-EM density with atomic model, showcasing the quality of the map. TM-helices are also shown for E and M.

Primer name	Sequence 5’ to 3’
DV2_mtE_H437E_F	AGATTGCTCCAAAGACTTGCTCGAGAGCCTTTCCTATAGAT
DV2_mtE_H437E_R	ATCTATAGGAAAGGCTCTCGAGCAAGTCTTTGGAGCAATCT
DV2_mtE_H437A_F	GATTGCTCCAAAGACTTGGGCGAGAGCCTTTCCTATAGAT
DV2_mtE_H437A_R	ATCTATAGGAAAGGCTCTCGCCCAAGTCTTTGGAGCAATC
DV2_mtE_G441Y_F	GCAGCTCCATAGATTGCATAAAAGACTTGGTGGAGAGCCTTTCC
DV2_mtE_G441Y_R	GGAAAGGCTCTCCACCAAGTCTTTTATGCAATCTATGGAGCTGC
hCMV-DV2-16681-5UTR-F	AGAGCTCGTTTAGTGAACCGAGTTGTTAGTCTACGTGGACCGACAAAGACAG
DV2-16681prM-start-R	CTCCGTTACGTGTGGTTAAATGGAA
DV2-16681prM-start-F	TTCCATTTAACCACACGTAACGGAG
DV2-16681-Eend_R	GGCCTGCACCATGACTCCCAAATACAG
DV2-16681-Eend_F	CTGTATTTGGGAGTCATGGTGCAGGCC
DV2-16681-6685-6709-R	CCAGTATTATTGAAGCTGCTATCCA
DV2-16681-6695-6724-F	CTTCAATAATACTGGAGTTTTTTCTCATAG
DV1-2-3-3UTR_R	AGAACCTGTTGATTCAACAGCACCATTCCATTTTCTGGCGTTCTGTGCCTGGAATGAT
DV1-2-3-3UTR-HDVAr-F	CTGTTGAATCAACAGGTTCTGGGTCGGCATGGCATCTCC
pcDNA-hCMV-R	CGGTTCACTAAACGAGCTCTGCTTATATAGACCTCCCACCG

Supplementary Table 1. Primers used in this study.
