# Supplementary material for: Flavivirus maturation leads to the formation of an occupied lipid pocket in the surface glycoproteins
Source: Nat Commun. 2021 Feb 23;12:1238. doi: 10.1038/s41467-021-21505-9 (PMC7902656; doi:10.1038/s41467-021-21505-9)
Supplement: Supplementary file 3 — Description of Additional Supplementary Files [file 41467_2021_21505_MOESM3_ESM.pdf]

Description of Additional Supplementary Files

File Name: Supplementary Movie 1
Description: **Cryo-EM map of prM_1E_1.** This video shows an overview of the quality of the prM_1E_1 cryo-EM map, obtained by localized reconstruction and focused refinement.

File Name: Supplementary Movie 2
Description: **Hypothetical model of the structural transition from immature spiky to smooth virion morphologies**. This video shows a hypothetical model of how the virus with immature trimeric prM_3E_3 surface spikes changes its conformation to the smooth state. These changes represent the pH transition along the secretory pathway, going from the ER (neutral pH) to the late Golgi (acidic pH). The video highlights how the furin recognition motif is concealed in the spiky virus, and then becomes exposed at the surface in our model of the smooth, pr-bound virus.
