# Supplementary material for: Flavivirus maturation leads to the formation of an occupied lipid pocket in the surface glycoproteins
Source: Nat Commun. 2021 Feb 23;12:1238. doi: 10.1038/s41467-021-21505-9 (PMC7902656; doi:10.1038/s41467-021-21505-9)
Supplement: Supplementary file 6 — Reporting Summary [file 41467_2021_21505_MOESM6_ESM.pdf]

nature research

Corresponding author(s): Jonathan M Grimes

Last updated by author(s): Jan 10, 2021

Reporting Summary

Nature Research wishes to improve the reproducibility of the work that we publish. This form provides structure for consistency and transparency in reporting. For further information on Nature Research policies, see our Editorial Policies and the Editorial Policy Checklist.

Statistics

For all statistical analyses, confirm that the following items are present in the figure legend, table legend, main text, or Methods section.

n/a	Confirmed	
☒	☐	The exact sample size (*n*) for each experimental group/condition, given as a discrete number and unit of measurement
☒	☐	A statement on whether measurements were taken from distinct samples or whether the same sample was measured repeatedly
☒	☐	The statistical test(s) used AND whether they are one- or two-sided<br>*Only common tests should be described solely by name; describe more complex techniques in the Methods section.*
☒	☐	A description of all covariates tested
☒	☐	A description of any assumptions or corrections, such as tests of normality and adjustment for multiple comparisons
☒	☐	A full description of the statistical parameters including central tendency (e.g. means) or other basic estimates (e.g. regression coefficient) AND variation (e.g. standard deviation) or associated estimates of uncertainty (e.g. confidence intervals)
☒	☐	For null hypothesis testing, the test statistic (e.g. *F*, *t*, *r*) with confidence intervals, effect sizes, degrees of freedom and *P* value noted<br>*Give P values as exact values whenever suitable.*
☒	☐	For Bayesian analysis, information on the choice of priors and Markov chain Monte Carlo settings
☒	☐	For hierarchical and complex designs, identification of the appropriate level for tests and full reporting of outcomes
☒	☐	Estimates of effect sizes (e.g. Cohen's *d*, Pearson's *r*), indicating how they were calculated

Our web collection on statistics for biologists contains articles on many of the points above.

Software and code

Policy information about availability of computer code

Data collection	SerialEM 3.7
Data analysis	MotionCor2-1.1.0, Gctf-v.1.06, CTFFIND4, cryoSPARC v.2.12.0, Ethan1.2, RELION3.1, Scipion v.2.0, PHENIX v.1.17.1, Coot v.0.8.9.2, MolProbity v.4.2

For manuscripts utilizing custom algorithms or software that are central to the research but not yet described in published literature, software must be made available to editors and reviewers. We strongly encourage code deposition in a community repository (e.g. GitHub). See the Nature Research guidelines for submitting code & software for further information.

Data

Policy information about availability of data

All manuscripts must include a data availability statement. This statement should provide the following information, where applicable:

- Accession codes, unique identifiers, or web links for publicly available datasets
- A list of figures that have associated raw data
- A description of any restrictions on data availability

All data are available from the corresponding authors and/or are included in the manuscript. Cryo-EM density maps and atomic coordinates of models have been deposited in the Electron Microscopy Data Bank and the Protein Data Bank (PDB), respectively. Atomic coordinates are deposited under the PDB accession codes PDB-ID 6ZQU (mature DENV2, icosahedral reconstruction), PDB-ID 6ZQV (mature SPOV, icosahedral reconstruction), PDB-ID 6ZQJ (immature SPOV trimeric spike, localized reconstruction), PDB-ID 6ZQI (immature prM1E1 monomer, localized reconstruction), and PDB-ID 6ZQW (immature SPOV, icosahedral reconstruction). Cryo-EM maps are deposited under the EMD accession codes EMD-11370 (mature DENV2, icosahedral reconstruction), EMD-11371 (mature SPOV, icosahedral reconstruction), EMD-11366 (immature SPOV trimeric spike, localized reconstruction), EMD-11364 (immature prM1E1 heterodimer, localized reconstruction), and EMD-11372 (immature SPOV, icosahedral reconstruction).

Field-specific reporting

Please select the one below that is the best fit for your research. If you are not sure, read the appropriate sections before making your selection.

- [x] Life sciences
- [ ] Behavioural & social sciences
- [ ] Ecological, evolutionary & environmental sciences

For a reference copy of the document with all sections, see nature.com/documents/nr-reporting-summary-flat.pdf

Life sciences study design

All studies must disclose on these points even when the disclosure is negative.

Sample size	No sample size calculation was performed as this study purely made use of in-vitro data. No ex-vivo experiments were performed. For the cryo-EM data set, as many images were collected as was possible in the allocated measurement time at the Electron Bio-Imaging Centre (eBIC) UK national facility.
Data exclusions	None
Replication	2 independent experiments. All replications were successful.
Randomization	We were not carrying out randomised trials. This is a structure-function analysis. Randomization was not relevant, as cryo-EM images were collected automatically, precluding selection bias.
Blinding	This is a structure-function analysis and in-vivo studies were not carried out. Blinding was not relevant to the study as cryo-EM images were acquired automatically.

Reporting for specific materials, systems and methods

We require information from authors about some types of materials, experimental systems and methods used in many studies. Here, indicate whether each material, system or method listed is relevant to your study. If you are not sure if a list item applies to your research, read the appropriate section before selecting a response.

Materials & experimental systems

n/a	Involved in the study	
	x	Antibodies
	x	Eukaryotic cell lines
x		Palaeontology and archaeology
x		Animals and other organisms
x		Human research participants
x		Clinical data
x		Dual use research of concern

Methods

n/a	Involved in the study	
x		ChIP-seq
x		Flow cytometry
x		MRI-based neuroimaging

Antibodies

Antibodies used	Antibody 4G2 is a gift from from Dr Malasit, Mahidol University, Thailand.<br><br>Antibody 749B12 is an antibody produced in-house and previously reported by Dejnirattisai et al., 2015 (Pubmed ID:25501631).<br><br>Alkaline-phosphatase conjugated anti-human IgG is commercially available from Sigma (A9544)
Validation	4G2 is a mouse antibody with group cross-reactivity to all flaviviruses and has been described, validated, and used for the detection of flavivirus E extensively in the literature, for instance in Summers et al., 1989 (Pubmed ID: 2543161) or Aubry et al., 2016 (Pubmed ID: 26283013).<br><br>Antibody 749B12 was validated in Dejnirattisai et al., 2015 (Pubmed ID:25501631). The study demonstrated its cross-reactive binding to Dengue virus serotypes 1 to 4.<br><br>Alkaline-phosphatase conjugated anti-human from Sigma (https://www.sigmaaldrich.com/catalog/product/sigma/a9544?lang=en&region=GB) is commercially available and has been validated by the vendor. Validation data are available on the vendor's website.

Eukaryotic cell lines

Policy information about cell lines

Cell line source(s)	Human embryonic kidney HEK 293T cells were commercially obtained from ATCC (CRL-3216). C6/36 were a gift from from Dr Malasit, Mahidol University, Thailand.
Authentication	None of the cell lines have been authenticated
Mycoplasma contamination	Yes, all cell lines and viral stocks were free from mycoplasma
Commonly misidentified lines (See ICLAC register)	No commonly misidentified cell lines were used.
